# Clinical Impact of Routine HBV Screening in Oncologic Patients Prior to Chemotherapy

**DOI:** 10.3390/jcm15072757

**Published:** 2026-04-06

**Authors:** Husam Abu Sini, Natali Shirron, Michael Litvak, Roni Nasser, Fadi Abu Baker, Rawi Hazzan, Nissim Haim, Tarek Saadi

**Affiliations:** 1 Bruce Rappaport Faculty of Medicine, Technion-Israel Institute of Technology, Haifa 3200003, Israel; h_abusini@rambam.health.gov.il (H.A.S.); m_litvak@rambam.health.gov.il (M.L.); r_nasser@rambam.health.gov.il (R.N.); n_haim@rambam.health.gov.il (N.H.); 2 Division of Oncology, Rambam Health Care Campus, Haifa 3109601, Israel; n_shirron@rambam.health.gov.il; 3 Liver Unit, Rambam Health Care Campus, Haifa 3109601, Israel; 4 Department of Gastroenterology, Rambam Health Care Campus, Haifa 3109601, Israel; 5 Internal Medicine B, Rambam Health Care Campus, Haifa 3109601, Israel; 6 Department of Gastroenterology and Hepatology, Hillel Yaffe Medical Center, Hadera 38100, Israel; 7 Clalit Health Services, Afula 1834111, Israel; rawihazzan1@gmail.com; 8 Azrieli Faculty of Medicine, Bar-Ilan University, Safed 1311502, Israel

**Keywords:** HBV, oncology patients, chemotherapy, antiviral therapy

## Abstract

**Background:** Hepatitis B virus (HBV) reactivation in oncology patients receiving chemotherapy can cause severe hepatitis, including hepatic failure and death. Universal HBV screening before chemotherapy initiation can reduce HBV-related morbidity; however, screening practices vary widely, and guideline recommendations continue to evolve. **Objective:** The aim of this study was to evaluate the implementation of universal HBV screening in oncology patients and its effectiveness in identifying active infection, prior exposure, and individuals at risk for HBV reactivation. **Methods:** We implemented universal HBV screening at Rambam Health Care Campus in January 2018 for all patients initiating chemotherapy. This retrospective cohort study analyzed 1614 oncology patients who underwent chemotherapy between January 2018 and April 2024. Screening included testing for HBsAg, anti-HBc, and anti-HBs serology. HBV DNA testing was performed in patients with positive HBsAg and/or anti-HBc serology. Patients with known HBsAg positivity or those already receiving antiviral therapy were excluded. **Results:** Of the screened patients, 16 (1.0%) were HBsAg-positive, and 134 (8.3%) were HBsAg-negative/anti-HBc-positive. Detectable HBV DNA was identified in four patients (3%) within the latter group. One additional patient was classified as high risk for HBV reactivation based on the planned chemotherapy regimen. Overall, 21 patients met criteria for prophylactic antiviral therapy; however, prophylaxis was administered to only 17 patients. Notably, when applying the 2015 ASCO guidelines, only a single patient within the subgroup of HBsAg-negative, anti-HBc-positive, and HBV DNA-negative patients would have qualified for HBV serologic screening based on chemotherapy-related risk alone. **Conclusions:** Universal HBV screening prior to chemotherapy enables the identification of patients with active or prior HBV infection who would not have been detected using risk-based screening strategies alone. Our findings further support the implementation of universal HBV screening in oncology settings to prevent HBV reactivation and its potentially severe consequences.

## 1. Introduction

Hepatitis B virus (HBV) infection is a major global health concern that is associated with acute and chronic liver disease, cirrhosis, and hepatocellular carcinoma. Chronic HBV carriage, often asymptomatic, remains prevalent in certain populations, especially among individuals born before the implementation of universal HBV vaccination in Israel in 1992 or those originating from highly endemic countries [1,2].

In Israel, the prevalence of HBV varies by ethnicity, at approximately 1.5% among Jews and 3–4% among non-Jewish populations, with rates as high as 10–20% among certain immigrant groups from Asia or Africa [3,4]. These epidemiological disparities highlight the importance of risk-based and potentially universal screening strategies in at-risk populations.

Cancer patients undergoing chemotherapy are at increased risk of HBV reactivation due to treatment-related immunosuppression. Such reactivation can result in acute hepatitis, liver failure, and potentially death [5,6].

The American Society of Clinical Oncology (ASCO) currently recommends that all patients with cancer undergoing systemic anticancer therapy should be screened for HBV using three serologic tests: hepatitis B surface antigen (HBsAg), total or IgG hepatitis B core antibody (anti-HBc), and hepatitis B surface antibody (anti-HBs). Screening should occur prior to or at the beginning of therapy, but the initiation of cancer treatment should not be delayed pending results. Patients identified as HBsAg-positive (chronic HBV) or HBsAg-negative/anti-HBc-positive (past HBV infection) require further risk assessment and management. Antiviral prophylaxis is strongly recommended for chronic HBV patients during and for at least 12 months after systemic therapy. For those with past HBV infection, antiviral prophylaxis is indicated if receiving high-risk regimens (e.g., anti-CD20 monoclonal antibodies, stem cell transplantation), while close monitoring is appropriate for lower-risk regimens [7].

The American Association for the Study of Liver Diseases (AASLD) also recommends HBV screening (HBsAg and anti-HBc) for all patients prior to immunosuppressive or cytotoxic therapy, including cancer chemotherapy. Prophylactic antiviral therapy should be initiated in HBsAg-positive and anti-HBc-positive patients before or at the onset of immunosuppressive therapy, and continued for at least 6 months after therapy (12 months for anti-CD20 therapies). For HBsAg-negative/anti-HBc-positive patients, monitoring is appropriate except in high-risk settings, where prophylaxis is recommended [8].

The European Association for the Study of the Liver (EASL) and the European Society for Medical Oncology (ESMO) similarly endorse for HBV screening in patients undergoing cancer therapy, with ESMO specifically recommending screening for patients with lymphoma and prophylactic antiviral therapy for those with positive serology, including occult carriers [9].

Previous ASCO guidelines evolved over three iterations (2010, 2015, and 2020), marking a paradigm shift from risk-based to universal screening. The initial 2010 ASCO guideline suggested that clinicians screen only those at high risk for chronic HBV infection or if highly immunosuppressive therapies, such as rituximab or hematopoietic cell transplantation, were planned. This represented a selective, risk-adaptive approach focused on high-risk populations and high-risk therapies [10].

The 2015 update introduced a more structured risk-adaptive strategy. Medical providers were advised to screen patients for HBV infection before anti-CD20 therapy or transplantation, alongside those with known risk factors. Screening comprised both hepatitis B surface antigen (HBsAg) and hepatitis B core antibody (anti-HBc), using either total anti-HBc or anti-HBc immunoglobulin G (not IgM) [11].

However, the 2015 guideline explicitly stated that for patients who neither had HBV risk factors nor anticipated cancer therapy associated with a high risk of reactivation, current evidence did not support HBV screening before initiation of cancer therapy. Notably, two panel members offered a minority viewpoint advocating for universal HBsAg and selective anti-HBc testing, predicting the eventual shift in recommendations [11].

This study aims to evaluate the benefits of routine HBV screening among oncology patients prior to the initiation of chemotherapy at the Rambam Health Care Campus (RHCC). The objective is to identify carriers and previously exposed individuals to facilitate prophylactic antiviral treatment and prevent HBV reactivation during therapy.

## 2. Methods

This retrospective cohort study included adult patients with solid malignancies who were admitted and hospitalized for chemotherapy initiation at Rambam Health Care Campus between 1 January 2018, and 30 April 2025. The study protocol was approved by the institutional ethics committee and conducted in accordance with the Declaration of Helsinki.

Clinical, laboratory, and demographic data were automatically extracted from a computerized hospital database directly linked to the electronic medical record (EMR) system. Data extraction was performed using predefined queries to ensure completeness and minimize manual entry errors. Collected variables included patient demographics (age, sex, and ethnicity), oncologic diagnosis, chemotherapy regimen, HBV serologic markers (HBsAg, anti-HBc, anti-HBs), HBV DNA results, and antiviral treatment status. The distribution of HBV markers and clinical characteristics was analyzed across patient subgroups. Laboratory platforms and kits used at the Rambam Health Care Campus virology laboratory included the Alinity i HBsAg Qualitative ABBOTT Wiesbaden Germany for HBsAg; the Xpert^®^ HBV Viral Load Cepheid, Sunnyvale, CA, USA (LLoQ of 5.99 IU/mL) for HBV DNA; and the Alinity i Anti-HBc ABBOTT for Anti-HBc.

In January 2018, RHCC implemented a universal HBV screening policy for all patients initiating chemotherapy. Under this protocol, patients underwent routine serologic testing for HBsAg and anti-HBc before commencing treatment. Screening included serologic testing for hepatitis B surface antigen (HBsAg) and hepatitis B core antibody (anti-HBc). HBV DNA testing by polymerase chain reaction (PCR) was performed in all patients with positive HBsAg and/or anti-HBc serology.

Patients who were HBsAg-positive received prophylactic antiviral therapy with either entecavir or tenofovir before starting chemotherapy. Patients who were HBsAg-negative but anti-HBc-positive underwent HBV DNA testing. Antiviral prophylaxis was initiated in those with detectable HBV DNA (occult HBV infection), while patients with undetectable HBV DNA were monitored during chemotherapy. In accordance with institutional protocols, antiviral prophylaxis is typically continued for at least 12 months after completion of chemotherapy, and patients are generally monitored for an additional year after discontinuation of antiviral therapy to assess for possible reactivation. Furthermore, patients with evidence of prior HBV exposure (HBsAg-negative/anti-HBc-positive) who did not receive antiviral therapy are routinely followed every 3 months during chemotherapy and for at least 12 months after completion of chemotherapy to monitor for reactivation.

Patients with known HBsAg positivity or those already receiving antiviral therapy were excluded from the study.

In addition, treatment decisions were reviewed according to chemotherapy-related immunosuppression risk. Prophylactic antiviral therapy was considered for selected patients receiving highly immunosuppressive chemotherapy regimens, irrespective of baseline HBV DNA results.

The definition of hepatitis B virus (HBV) reactivation during chemotherapy treatment is an abrupt increase in HBV replication in a patient with previously inactive or resolved HBV infection, typically evidenced by a rise in serum HBV DNA levels, often accompanied by an increase in serum alanine aminotransferase (ALT) levels. Clinical presentations vary from asymptomatic to severe, potentially life-threatening hepatitis flares. Aligned with international guidelines, patients who were HBsAg-positive or had detectable HBV DNA were generally managed with antiviral prophylaxis; therefore, classical reactivation events would be expected primarily among patients who were HBsAg-negative and anti-HBc-positive [9]. In this context, reactivation was considered clinically when patients demonstrated either newly detectable HBV DNA (virologic reactivation) or HBsAg seroreversion (seroconversion from negative to positive), as documented in the medical record. Given the study’s retrospective nature, reactivation assessment relied on physician-documented clinical evaluation rather than prospectively applied biochemical or virologic criteria.

### Statistical Analysis

Descriptive statistics were used to summarize demographic, clinical, and laboratory characteristics of the study population. Continuous variables were presented as mean ± standard deviation (SD). Categorical variables were reported as frequencies and percentages. Continuous variables were compared using the Mann–Whitney U test and categorical variables using the chi-square test. Multivariable logistic regression was performed to identify independent predictors of HBV exposure, with variables selected by backward elimination (*p* < 0.05). Model performance was assessed using the area under the receiver operating characteristic curve (AUC-ROC). A *p*-value <0.05 was considered statistically significant. Analyses were performed using Python 3.12 with statsmodels and scipy packages.

## 3. Results

### 3.1. Patient Characteristics

Between January 2018 and April 2025, 1614 patients with malignancies underwent routine HBV screening prior to chemotherapy initiation at Rambam Health Care Campus (RHCC). A total of 5209 records were retrieved from the database, corresponding to 1614 unique patients (Table 1). The cohort included 983 males (60.9%) and 631 females (39.1%). The mean age at the reference laboratory test was 63.6 years (SD 13.4), with a median age of 65.8 years (interquartile range [IQR] 16.1; range 18.1–94.5). The total accumulated follow-up across the cohort was 48 patient-years, with a calculated median follow-up of 0 months (range 0–63.6 months). Most patients were of Jewish ethnicity (57.1%), followed by Arab Muslim (16.0%). All patients were managed within the oncology division. The most prevalent diagnosis was gastrointestinal malignancy (46.2%), followed by lung cancer (13.7%), genitourinary malignancies (7.2%), and sarcoma (6.5%). Other malignancies included neuro-oncologic (3.3%), breast (3.2%), head and neck (2.4%), gynecologic (1.5%), and hematologic (1.7%) cancers.

Chemotherapy regimens were heterogeneous and reflected the underlying disease distribution. Fluoropyrimidine-based combinations (including oxaliplatin and irinotecan) and platinum-based protocols were the most frequently administered treatments.

### 3.2. HBV Prevalence and Prophylaxis

**Figure 1:**
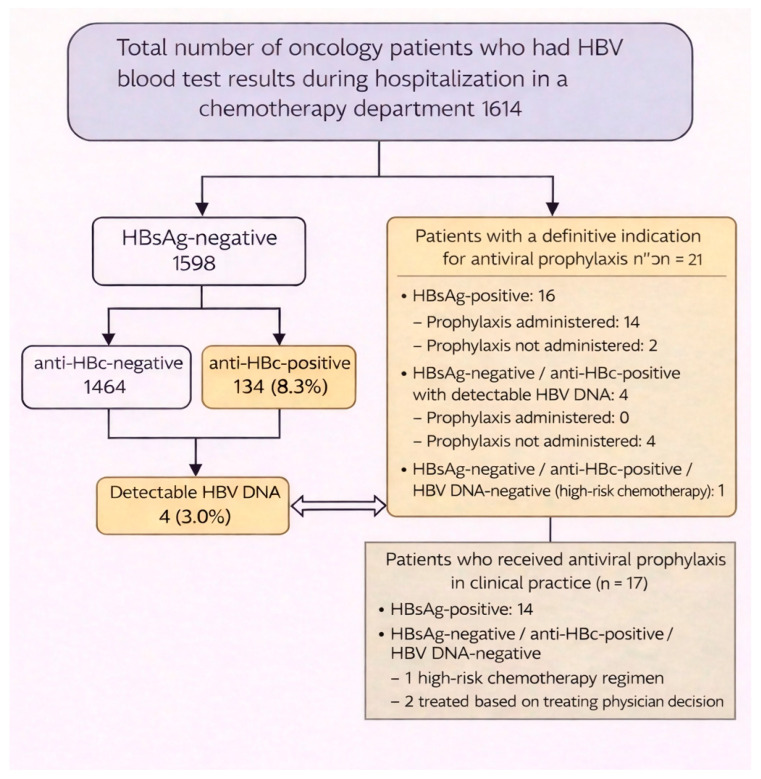
Flow diagram showing HBV screening results and antiviral prophylaxis decision-making in 1614 oncology patients. Upper panel shows guideline-based indications (n = 21); lower panel shows actual prophylaxis administration (n = 17). The gap between indications and administration includes 7 patients with indications who did not receive prophylaxis (2 HBsAg-positive patients who declined treatment, 4 DNA-positive patients with very low viral loads, and 1 high-risk chemotherapy patient) and 3 patients who received prophylaxis beyond strict guidelines (anti-HBc-positive/DNA-negative patients with high-risk chemotherapy). HBsAg, hepatitis B surface antigen; anti-HBc, hepatitis B core antibody; DNA, HBV DNA.

Of the 1614 screened patients, 16 (1.0%) were positive for hepatitis B surface antigen (HBsAg) and were classified as having chronic HBV infection. Of these, 14 patients (87.5%) received antiviral prophylaxis prior to chemotherapy initiation, while two patients declined prophylactic therapy (Figure 1). Among the remaining HBsAg-negative patients, 134 (8.3%) were anti-hepatitis B core antibody (anti-HBc)-positive, indicating prior HBV exposure. Among HBsAg-negative patients who were anti-HBc-positive, detectable HBV DNA was identified in four patients (3%), consistent with occult HBV infection. All four patients had very low viral loads (<10 IU/mL), below the threshold typically requiring prophylaxis; therefore, none of these patients received prophylactic antiviral therapy, and instead underwent clinical monitoring.

The remaining 130 anti-HBc-positive patients had undetectable HBV DNA. Prophylactic antiviral therapy was initiated in three of these patients (2.3%) at the discretion of the treating physician, one of whom was considered high-risk due to the specific chemotherapy regimen. The remaining 127 patients underwent quarterly monitoring of liver enzymes, HBsAg, and HBV DNA.

Overall, 21 patients met guideline-based indications for prophylactic antiviral therapy: 16 HBsAg-positive patients, 4 anti-HBc-positive patients with detectable HBV DNA, and 1 anti-HBc-positive with undetectable HBV DNA patient scheduled to receive highly immunosuppressive chemotherapy. In clinical practice, 17/21 patients (81.0%) received prophylaxis. The four patients with indications who did not receive prophylaxis included two who declined treatment and two who were managed expectantly due to very low viral loads. Conversely, three patients received prophylaxis based on clinical assessment despite not meeting strict criteria (Figure 1). Among the 17 treated patients, 14 received tenofovir, two received entecavir, and one received lamivudine.

No cases of HBV reactivation were observed during the follow-up period (median 0 months; range 0–63.6). However, it is important to emphasize that this study was not powered nor designed to evaluate reactivation risk. The limited systematic follow-up represents a significant limitation in assessing true reactivation rates.

### 3.3. Predictors of HBV Exposure

Serologic evidence of HBV exposure (HBsAg-positive and/or anti-HBc-positive) was found in 150 patients (9.3%). Compared to non-exposed patients, the HBV-exposed group was significantly older (68.6 ± 8.8 vs. 63.0 ± 13.7 years, *p* < 0.001) and predominantly male (70.7% vs. 59.9%, *p* = 0.013). Mortality rates were comparable between the two groups (26.7% each; *p*-value = 1). Cancer type distribution was also similar, with gastrointestinal malignancies predominating in both cohorts (46.7% vs. 46.2%, *p* = 0.977) (Table 2).

Multivariable logistic regression identified three independent demographic predictors of HBV exposure (Table 3). Age was the strongest predictor (adjusted odds ratio [aOR] 1.04 per year, 95% CI 1.03–1.06, *p* < 0.001). Male gender was associated with increased odds of exposure (aOR 1.60, 95% CI 1.11–2.32, *p* = 0.014), while Jewish ethnicity was inversely associated with exposure (aOR 0.66, 95% CI 0.47–0.93, *p* = 0.018). No specific cancer type showed a significant independent association with HBV exposure after adjusting for demographic factors.

## 4. Discussion

Our findings underscore the clinical role of routine hepatitis B virus (HBV) serologic screening for oncology patients prior to chemotherapy. HBV reactivation remains a potentially severe and occasionally fatal complication of immunosuppressive therapy; early identification of at-risk patients enables timely antiviral intervention, significantly reducing associated morbidity and mortality.

While the HBsAg positivity rate in our cohort was 1.0%, partially reflecting the exclusion of previously known carriers, the identification of occult viremia in HBsAg-negative/anti-HBc-positive patients highlights the limitations of risk-based screening. Notably, four patients with occult HBV infection (HBsAg-negative but anti-HBc-positive were found to have detectable HBV DNA) would have been overlooked by selective screening approaches despite having a clear clinical indication for prophylaxis. This discrepancy aligns with the evolution of ASCO guidelines; our data suggests that reliance on 2015-era selective screening would have resulted in substantial underdiagnosis.

A key observation in our study was the “implementation gap” in clinical practice: 4 out of 21 patients (19.0%) with guideline-based indications for prophylaxis did not receive it. While no reactivation events were observed during the follow-up period, this should be interpreted with caution. HBV reactivation is often unpredictable and may manifest later in the disease course or under more intensive immunosuppressive regimens. This highlights an urgent need for oncologists to systematically review viral serology before initiating treatment to ensure at-risk patients are not overlooked.

Furthermore, 134 patients (8.3%) in our cohort demonstrated prior HBV exposure (anti-HBc-positive). Although most were non-viremic at baseline, the risk of reactivation persists during treatment. In settings where close longitudinal monitoring is unfeasible, international guidelines advocate for universal prophylaxis in this subgroup. Additionally, our use of anti-HBs testing identified non-immune patients who could benefit from vaccination, providing a broader strategy for infection control in the oncology setting.

The shift toward universal screening in recent years was necessitated by evidence that risk-based assessments are often inaccurate or omitted in busy clinical environments. Studies by Ramsey et al. and Hwang et al. demonstrated that a significant portion of HBV-positive patients lack traditional risk factors, making selective screening both inefficient and impractical [12,13]. Our results reinforce this paradigm shift, proving that a universal approach is more reliable for identifying occult carriers who otherwise remain “invisible” to the clinician.

A distinct contribution of this study is the emphasis on HBV DNA assessment in HBsAg-negative/anti-HBc-positive patients. While major professional guidelines do not yet mandate routine DNA testing for all such patients, our data shows that a subset exhibits detectable viremia despite negative surface antigen results. We argue that these patients face an elevated risk and should be prioritized for prophylaxis. Moreover, since anti-HBc titers can wane over time, leading to potential false negatives, we suggest that assessing HBeAg/anti-HBe and reviewing historical serology may further refine risk stratification.

A critical finding of this study centers on the diagnostic gap in current professional guidelines. While major oncology and hepatology societies primarily rely on HBsAg and anti-HBc serology to guide prophylactic decisions, our data suggests that these markers alone may be insufficient. Specifically, we observed that a subset of HBsAg-negative/anti-HBc-positive patients (n = 4, 3%) exhibited detectable HBV DNA.

Despite being HBsAg-negative, these individuals harbor active viral replication, placing them at a significantly higher risk for reactivation compared to non-viremic exposed patients. Current clinical guidelines generally do not mandate routine HBV DNA testing for this HBsAg-negative/anti-HBc-positive subgroup, potentially missing those who require immediate prophylaxis. This observation aligns with research in hematologic malignancies, such as the work published in Hepatology, which emphasized the necessity of molecular testing to accurately stratify risk in immunosuppressed populations [14]. Our findings extend this necessity to the solid tumor setting, advocating for the integration of baseline HBV DNA quantification into standard screening protocols for all patients with prior HBV exposure.

In addition, in some patients with prior HBV exposure, anti-HBc antibody levels may decline over time, potentially resulting in a false-negative anti-HBc test [15]. To avoid missing such cases, we propose the routine assessment of HBeAg and anti-HBe as part of the baseline evaluation. Furthermore, the presence of anti-HBs positivity in patients without a history of HBV vaccination may serve as an additional clue to prior HBV exposure. Importantly, review of previous HBV serologic results, when available, may also be helpful in identifying such cases.

It is important to emphasize that patients without prior HBV exposure who are not vaccinated should be strongly recommended to receive HBV vaccination as early as possible before initiation of therapy [16].

From a health–economic perspective, universal HBV screening in patients initiating chemotherapy has generally been shown to be cost-effective or cost-saving in settings with a meaningful background prevalence of HBV and substantial risk of reactivation, particularly when antiviral prophylaxis prevents treatment interruptions, liver failure, or death [17,18]. Earlier decision-analytic models in solid tumors and sarcomas have demonstrated that routine pre-chemotherapy screening plus prophylaxis falls within acceptable willingness-to-pay thresholds compared with no or selective screening, primarily driven by the relatively low cost of generic nucleos(t)ide analogs and the high cost of managing HBV reactivation events [18,19]. Our real-world data indicate that universal screening alone is insufficient. To realize the full economic and clinical benefits of such policies, systematic screening must be integrated with standardized HBV DNA testing for anti-HBc-positive patients and robust clinical pathways that ensure the consistent initiation and maintenance of prophylaxis [17].

**Figure 2:**
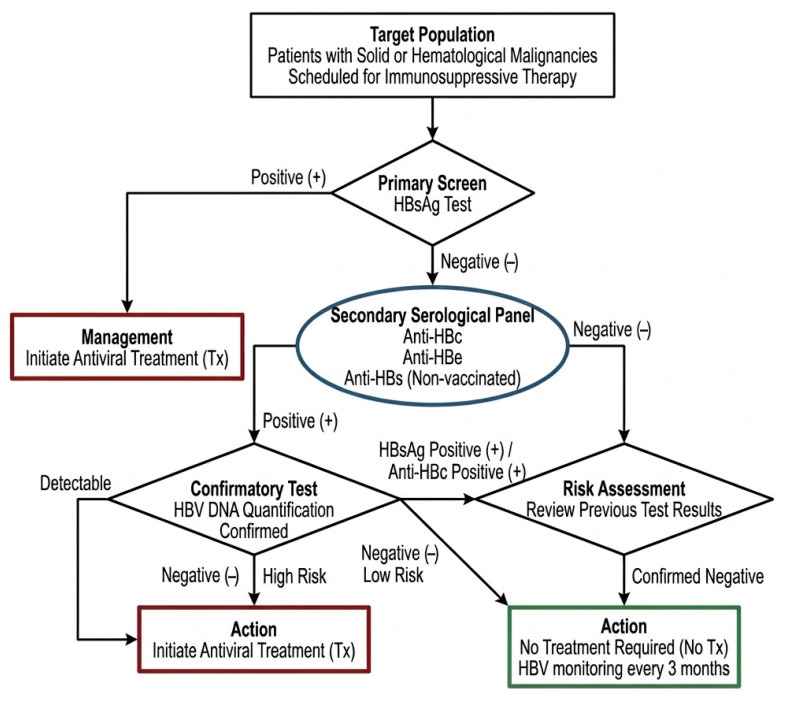
Proposed HBV screening and management algorithm. Patients starting immunosuppressive therapy are first screened for HBsAg; positive results require immediate antiviral treatment (Tx). Those who test negative undergo a secondary serological panel. If this panel is negative, no treatment is needed. If it is positive, HBV DNA quantification is performed: treatment is initiated if DNA is detectable or if high-risk factors are present. Conversely, no treatment is required for low-risk patients with undetectable DNA.

Taken together, we propose a comprehensive screening and management algorithm that incorporates all the parameters discussed above. This algorithm may be applicable to patients with solid tumors, as well as to patients with hematologic malignancies and those scheduled to receive immunosuppressive or immunomodulatory therapy (Figure 2).

Our study population included a relatively high proportion of non-Jewish patients and males, which may limit the generalizability of findings to the broader Israeli oncology population. However, this demographic variability also reflects real-world diversity and emphasizes the value of inclusive screening protocols.

Our findings strongly advocate for universal HBV screening as the standard of care for all oncology patients prior to chemotherapy, aligning with updated ASCO and EASL guidelines.

## 5. Conclusions

Our findings strongly support the implementation of universal HBV screening in all patients scheduled to receive immunosuppressive or immunomodulatory therapy, irrespective of cancer type or perceived risk factors. Routine serologic testing alone may be insufficient, as a clinically relevant subset of HBsAg-negative but anti-HBc-positive patients may harbor detectable HBV DNA and therefore remain at risk for viral reactivation. Accordingly, assessment of HBV DNA should be considered an integral component of the baseline evaluation in patients with evidence of prior HBV exposure. Importantly, careful review and interpretation of viral serology results by treating physicians is essential to ensure timely initiation of prophylactic antiviral therapy and to avoid missing patients with a clear indication for prevention. The adoption of a comprehensive and systematic screening strategy may substantially reduce the risk of HBV reactivation and its potentially severe clinical consequences in immunosuppressed patients.

## Figures and Tables

**Table 1:** Baseline characteristics of patients by HBV serologic and virologic status.

Characteristic	HBsAg-Positive	HBsAg−, Anti-HBc+, DNA+	HBsAg−, Anti-HBc+, DNA−	HBsAg-Negative, Anti-HBc-Negative
N (total patients) 1614 (100)	16 (1.0)	4	130 (8.1)	1464 (90.9)
N (%) of patients prescribed prophylactic antiviral therapy	14	0	3 (1 high risk)	0
Age (years), mean ± SD	61.6 ± 9.1	75.0 ± 5.2	69.3 ± 8.5	63.0 ± 13.7
Gender, n (%)				
Male	11 (68.8%)	4 (100.0%)	91 (70.0%)	877 (59.9%)
Female	5 (31.2%)	0 (0.0%)	39 (30.0%)	587 (40.1%)
Religion, n (%)				
Jewish	5 (31.2%)	2 (50.0%)	69 (53.1%)	845 (57.7%)
Non-Jewish/Unknown	11 (68.8%)	2 (50.0%)	61 (46.9%)	619 (42.3%)
Deceased, n (%)				
No	11 (68.8%)	4 (100.0%)	95 (73.1%)	1073 (73.3%)
Yes	5 (31.2%)	0 (0.0%)	35 (26.9%)	391 (26.7%)
Oncology Diagnosis Groups, n (%)				
Gastrointestinal (GI)	6 (37.5%)	2 (50.0%)	62 (47.7%)	676 (46.2%)
Lung	6 (37.5%)	1 (25.0%)	18 (13.9%)	196 (13.4%)
Genitourinary (GU)	0 (0.0%)	0 (0.0%)	6 (4.6%)	111 (7.6%)
Breast	1 (6.2%)	0 (0.0%)	5 (3.8%)	45 (3.1%)
Neuro-oncology	0 (0.0%)	0 (0.0%)	3 (2.3%)	51 (3.5%)
Head and neck	0 (0.0%)	0 (0.0%)	1 (0.8%)	38 (2.6%)
Gynecologic	0 (0.0%)	0 (0.0%)	1 (0.8%)	24 (1.6%)
Hematologic malignancy	0 (0.0%)	0 (0.0%)	1 (0.8%)	26 (1.8%)
Melanoma	0 (0.0%)	0 (0.0%)	1 (0.8%)	27 (1.9%)
Sarcoma	0 (0.0%)	0 (0.0%)	8 (6.2%)	96 (6.6%)
Other	3 (18.8%)	1 (25.0%)	24 (18.4%)	174 (11.9%)
Chemotherapy protocol, n (%)				
mFOLFOX6-based	3 (18.8%)	1 (25.0%)	49 (37.7%)	395 (27.0%)
Platinum + etoposide	1 (6.2%)	2 (50.0%)	12 (9.2%)	137 (9.4%)
FOLFIRINOX-based	0 (0.0%)	0 (0.0%)	11 (8.5%)	135 (9.2%)
Taxane + carboplatin	1 (6.2%)	0 (0.0%)	3 (2.3%)	48 (3.3%)
Immunotherapy-based	0 (0.0%)	0 (0.0%)	7 (5.4%)	37 (2.5%)
Other	11 (68.8%)	1 (25.0%)	48 (36.9%)	712 (48.6%)

Values are presented as n (%) for categorical variables and mean ± SD for continuous variables. HBsAg-negative/anti-HBc-positive patients are subdivided according to HBV DNA status for descriptive purposes. Subgroups are mutually exclusive. HBsAg = hepatitis B surface antigen; anti-HBc = hepatitis B core antibody; DNA = HBV DNA.

**Table 2:** Comparison of baseline characteristics between HBV-exposed and non-exposed patients.

Characteristic	HBV-Exposed (n = 150)	Non-Exposed (n = 1464)	*p*-Value
**Demographics**			
**Age (years), mean ± SD**	68.6 ± 8.8	63.0 ± 13.7	<0.001 *
**Male gender, n (%)**	106 (70.7)	877 (59.9)	0.013 *
**Jewish religion, n (%)**	76 (50.7)	845 (57.7)	0.115
**Clinical Outcomes**			
**Deceased, n (%)**	40 (26.7) **	391 (26.7)	0.004 *
**Cancer Type, n (%)**			
**Gastrointestinal**	70 (46.7)	676 (46.2)	0.977
**General oncology**	26 (17.3)	169 (11.5)	0.052
**Lung**	18 (12.0)	121 (8.3)	0.161
**Genitourinary**	6 (4.0)	111 (7.6)	0.148
**Sarcoma**	8 (5.3)	87 (5.9)	0.905

Values are presented as mean ± SD for continuous variables and n (%) for categorical variables. *p*-values were calculated using the Mann–Whitney U test for age and the chi-square test for categorical variables. HBV-exposed is defined as positive HBsAg and/or anti-HBc serology (with documented HBsAg-negative result). * Statistically significant at *p* < 0.05. ** Statistically significant at *p* < 0.01.

**Table 3:** Multivariable logistic regression analysis—independent predictors of HBV exposure.

Variable	Coefficient	Odds Ratio	95% CI	*p*-Value
**Demographics**				
**Age (per year increase)**	0.041	1.04	1.03–1.06	<0.001 *
**Male gender (vs. female)**	0.469	1.60	1.11–2.32	0.014 *
**Jewish religion (vs. non-Jewish)**	−0.421	0.66	0.47–0.93	0.018 *
**Cancer type (vs. other types)**				
**Gastrointestinal**	−0.037	0.96	0.66–1.40	0.848
**Lung**	0.208	1.23	0.69–2.19	0.479
**Genitourinary**	−0.787	0.46	0.19–1.10	0.080

* *p* < 0.05. Pseudo R^2^ = 0.044. Reference categories: female, non-Jewish, other cancers. CI = confidence interval.

## Data Availability

The data presented in this study are available on request from the corresponding author. The data are not publicly available due to privacy and ethical restrictions.
